# Angiotensin‐converting enzyme inhibitor induced cough compared with placebo, and other antihypertensives: A systematic review, and network meta‐analysis

**DOI:** 10.1111/jch.14695

**Published:** 2023-07-07

**Authors:** Yiyun Hu, Ling Liang, Shuang Liu, Janice Y. Kung, Hoan Linh Banh

**Affiliations:** ^1^ Department of Pharmacy Second Xiangya Hospital of Central South University Changsha China; ^2^ Department of Cardiology The Third Clinical Medical College, Fujian Medical University Fuzhou China; ^3^ Department of Cardiology The First Affiliated Hospital of Xiamen University Xiamen China; ^4^ Medical Affairs Management Department Second Xiangya Hospital of Central South University Changsha China; ^5^ University of Alberta, John W. Scott Health Sciences Library Edmonton Canada; ^6^ Faculty of Medicine and Dentistry Department of Family Medicine University of Alberta Edmonton Canada

**Keywords:** ACE inhibitors, angiotensin receptor blocker, calcium channel blockers, network meta‐analysis

## Abstract

Studies have shown that angiotensin converting enzyme inhibitors (ACEIs) are superior in primary and secondary prevention for cardiac mortality and morbidity to angiotensin receptor blocker (ARBs). One of the common side effects from ACEI is dry cough. The aims of this systematic review, and network meta‐analysis are to rank the risk of cough induced by different ACEIs and between ACEI and placebo, ARB or calcium channel blockers (CCB). We performed a systematic review, and network meta‐analysis of randomized controlled trials to rank the risk of cough induced by each ACEI and between ACEI and placebo, ARB or CCB. A total of 135 RCTs with 45,420 patients treated with eleven ACEIs were included in the analyses. The pooled estimated relative risk (RR) between ACEI and placebo was 2.21 (95% CI: 2.05–2.39). ACEI had more incidences of cough than ARB (RR 3.2; 95% CI: 2.91, 3.51), and pooled estimated of RR between ACEI and CCB was 5.30 (95% CI: 4.32–6.50) Moexipril ranked as number one for inducing cough (SUCRA 80.4%) and spirapril ranked the least (SUCRA 12.3%). The order for the rest of the ACEIs are as follows: ramipril (SUCRA 76.4%), fosinopril (SUCRA 72.5%), lisinopril (SUCRA 64.7%), benazepril (SUCRA 58.6%), quinapril (SUCRA 56.5%), perindopril (SUCRA 54.1%), enalapril (SUCRA 49.7%), trandolapril (SUCRA 44.6%) and, captopril (SUCRA 13.7%). All ACEI has the similar risk of developing a cough. ACEI should be avoided in patients who have risk of developing cough, and an ARB or CCB is an alternative based on the patient's comorbidity.

## 1 INTRODUCTION

Angiotensin converting enzyme inhibitors (ACEIs) plays an essential road in the prevention and treatment of cardiovascular diseases such as hypertension, coronary heart disease, heart failure, and other vascular diseases such as stroke.^1^ It is postulated that the activation of renin‐angiotensin‐aldosterone system (RAAS) leads to vasoconstriction, vascular smooth muscle and cardiac hypertrophy, and fibrosis.^2^ The consequences of the actions result in detrimental cardiac effects such as hypertension, myocardial infarction, and heart failure. The blockade of the RAAS using ACEIs has shown to reduce cardiac mortality and morbidity.^3^, ^4^, ^5^, ^6^ One of the common side effects from ACEI is dry cough.^7^ The incidence of cough associated with ACEI has been reported to be between 3.9% and 35%.^8^, ^9^ The exact mechanism of ACEI induced cough is unclear. It has been proposed that several mechanisms are involved. One study suggests that ACEI increase the sensitivity of the cough reflex.^10^ The most common suggested mechanism is that ACEI break down bradykinin and other inflammatory peptide in the lungs.^10^, ^11^ Another possible mechanism for ACEI‐induced cough may be associated with a defect in the degradation of bradykinin, which elevates the level of bradykinin.^12^ Frequently, when patients develop a cough from ACEI, clinicians switch ACEIs with an angiotensin receptor blocker (ARB). The use of ACEI or ARB is similar in the prevention of cardiovascular outcomes with respect to acute myocardial infarction, stroke and heart failure or hospitalization. Nevertheless, the use of ACEs compared to ARB is more effective in the reduction of total deaths and cardiovascular deaths.^13^ The objectives of this study are: (1) to complete a systematic review comparing ACEI with placebo, ARB, and calcium channel blockers (CCB) and cough; (2) to perform a network meta‐analysis to rank the risk of cough induced by different ACEIs (3) to perform a network meta‐analysis between placebo, ACEI, ARB and CCB to rank the risk of cough cause by each class of agents.

## 2 METHOD

The medical librarian (JYK) developed and executed comprehensive searches in Ovid MEDLINE, Ovid Embase, CINAHL, Scopus, and Cochrane Library (via Wiley) on March 21, 2022. To capture all relevant randomized controlled trials (RCTs) pertaining to ACE inhibitor induced cough in the general population, relevant keywords and controlled vocabulary were carefully selected. The search integrated a validated RCT filter for MEDLINE, which was subsequently adapted to other databases. Searches were limited to English language. Refer to appendix I for full‐text search strategies. The reporting of this network systematic review was guided by the standards of the Preferred Reporting Items for Systematic Review and Meta‐Analysis (PRISMA) Statement.^14^ This network meta‐analysis was registered on the PROSPERO website (CRD42021274659).

### 2.1 Data extraction and quality assessment

The references were independently reviewed by two authors (YYH, HLB). Disagreements were resolved by a third author (SL). The data were independently extracted by two authors (YYH, HLB). The data extracted include subject demographic characteristics, first author, journal and the year of publication, population, intervention, comparator, sample size, maximum ACEI dose, and incidence of cough. The meta‐analysis and network meta‐analysis consisted of only randomized controlled trials with the following inclusion criteria: (1) ACE inhibitor use, (2) placebo, or ARB, or CCB use, (3) incidence of cough. The excluded criteria are: (1) occurrence of cough before the trial; (2) having the past medical history of asthma.

### 2.2 Statistical analysis

#### 2.2.1 Network meta‐analysis

A network meta‐analysis was constructed to build the connective relationship within multi‐arms and between studies. The indirect evaluations of cough risk ratios (RRs) for different single ACEI treatments that had not been compared head‐to‐head directly were determined. By entering every event arm data and total numbers in the Stata software^®^, a network map of these connections and a network forest of estimated RRs were created. In addition, cough risks induced by different ACEIs were ranked according to the surface under the cumulative ranking curve (SUCRA). SUCRA values range from 0% to 100%. The higher the SUCRA value, and the closer to 100%, the higher the likelihood that ACEI is in the top rank inducing cough; the closer to 0 the SUCRA value, the more likely that ACEI is in the bottom rank inducing cough.

The process of network meta‐analysis includes using the global inconsistency test and node‐splitting approach to check for inconsistency to justify by using combination of direct and indirect evidence. Normally, the random model in the consistency test is used. If no heterogeneity was found in the inconsistency test, the fixed model was used to perform the consistency test. Publication bias was estimated by comparison‐adjusted funnel plots. A two‐tailed *p*‐value < .05 was considered statistically significant. All the statistical analyses were performed in Stata 14.1 (Stata Corp, College Station, TX).

## 3 RESULTS

**FIGURE 1:**
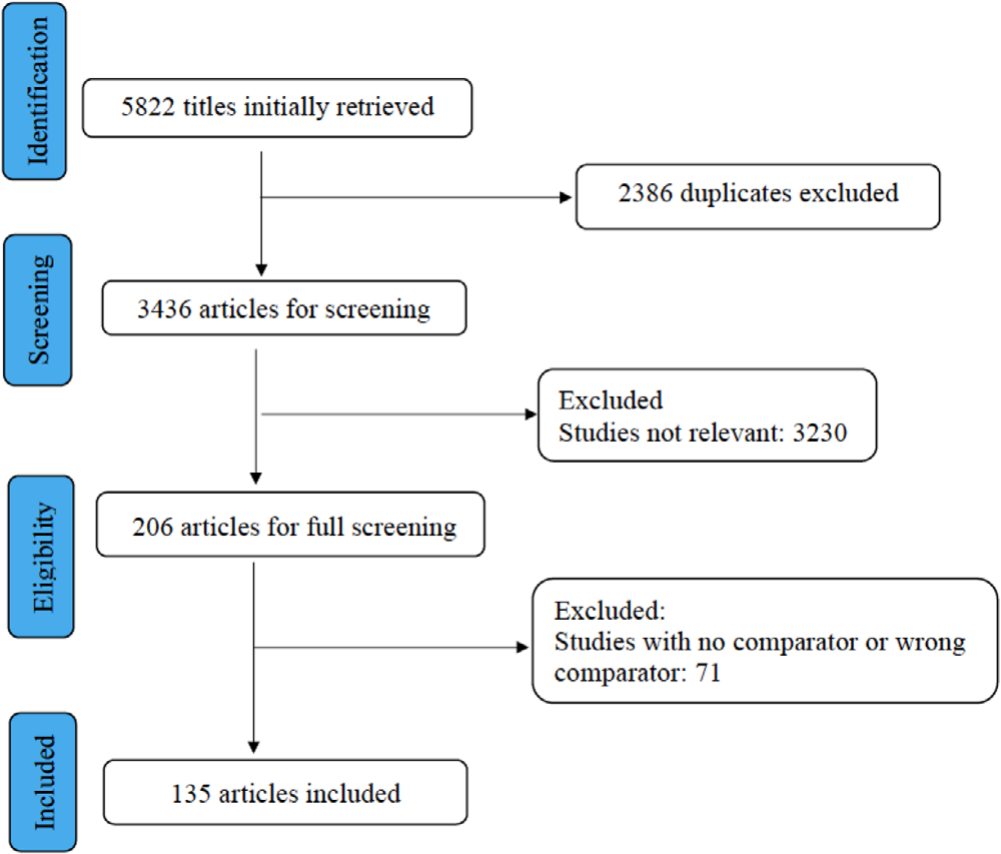
PRISMA diagram.

The complete search strategies are summarized in appendix I. A total of 5822 results were retrieved and after removing duplicates, 3436 unique results remained for the initial title and abstract screening in Covidence, a web‐based tool (www.covidence.org). In addition to subscription databases, the research team reviewed the first 200 results from Google Scholar. Bibliographies from included studies were also reviewed. A total of 206 studies were identified. After screening the full text, 135 RCTs with 45,420 participants treated with eleven ACEIs were included. Figure 1. The age of the participants ranged from 7 to 78 years old. The studies included participants from a wide range of medical conditions including hypertension, transient ischemic attack, coronary artery disease, proteinuria, heart failure, and organ transplant. The basic characteristics of the studies are in Table 1.^15^, ^16^, ^17^, ^18^, ^19^, ^20^, ^21^, ^22^, ^23^, ^24^, ^25^, ^26^, ^27^, ^28^, ^29^, ^30^, ^31^, ^32^, ^33^, ^34^, ^35^, ^36^, ^37^, ^38^, ^39^, ^40^, ^41^, ^42^, ^43^, ^44^, ^45^, ^46^, ^47^, ^48^, ^49^, ^50^, ^51^, ^52^, ^53^, ^54^, ^55^, ^56^, ^57^, ^58^, ^59^, ^60^, ^61^, ^62^, ^63^, ^64^, ^65^, ^66^, ^67^, ^68^, ^69^, ^70^, ^71^, ^72^, ^73^, ^74^, ^75^, ^76^, ^77^, ^78^, ^79^, ^80^, ^81^, ^82^, ^83^, ^84^, ^85^, ^86^, ^87^, ^88^, ^89^, ^90^, ^91^, ^92^, ^93^, ^94^, ^95^, ^96^, ^97^, ^98^, ^99^, ^100^, ^101^, ^102^, ^103^, ^104^, ^105^, ^106^, ^107^, ^108^, ^109^, ^110^, ^111^, ^112^, ^113^, ^114^, ^115^, ^116^, ^117^, ^118^, ^119^, ^120^, ^121^, ^122^, ^123^, ^124^, ^125^, ^126^, ^127^, ^128^, ^129^, ^130^, ^131^, ^132^, ^133^, ^134^, ^135^, ^136^, ^137^, ^138^, ^139^, ^140^, ^141^, ^142^, ^143^, ^144^, ^145^, ^146^, ^147^, ^148^, ^149^ A total of 44 RCT compared ACEI with placebo, 68 RCT with ARB, and 35 RCT with CCB. Various doses of ACEIs were used in the RCTs.

**TABLE 1:** basic characteristics of included studies.^15^, ^16^, ^17^, ^18^, ^19^, ^20^, ^21^, ^22^, ^23^, ^24^, ^25^, ^26^, ^27^, ^28^, ^29^, ^30^, ^31^, ^32^, ^33^, ^34^, ^35^, ^36^, ^37^, ^38^, ^39^, ^40^, ^41^, ^42^, ^43^, ^44^, ^45^, ^46^, ^47^, ^48^, ^49^, ^50^, ^51^, ^52^, ^53^, ^54^, ^55^, ^56^, ^57^, ^58^, ^59^, ^60^, ^61^, ^62^, ^63^, ^64^, ^65^, ^66^, ^67^, ^68^, ^69^, ^70^, ^71^, ^72^, ^73^, ^74^, ^75^, ^76^, ^77^, ^78^, ^79^, ^80^, ^81^, ^82^, ^83^, ^84^, ^85^, ^86^, ^87^, ^88^, ^89^, ^90^, ^91^, ^92^, ^93^, ^94^, ^95^, ^96^, ^97^, ^98^, ^99^, ^100^, ^101^, ^102^, ^103^, ^104^, ^105^, ^106^, ^107^, ^108^, ^109^, ^110^, ^111^, ^112^, ^113^, ^114^, ^115^, ^116^, ^117^, ^118^, ^119^, ^120^, ^121^, ^122^, ^123^, ^124^, ^125^, ^126^, ^127^, ^128^, ^129^, ^130^, ^131^, ^132^, ^133^, ^134^, ^135^, ^136^, ^137^, ^138^, ^139^, ^140^, ^141^, ^142^, ^143^, ^144^, ^145^, ^146^, ^147^, ^148^, ^149^

Author year	Journal	Study design	Demographics	Acei	Comparator	Maximum acei daily dose
Abdul‐Rahim AH, 2016	Eur Stroke J	Randomized, double blind	Population: MI Mean age range (yr): 44–66 Patients: (G1 = 4697, G2 = 4668, G3 = 4684)	G1: Captopril (236) G2: Captopril + Valsartan (217)	G3: Valsartan^81^	40 mg
Agabiti‐Rosei E, 1999	Eur J Clin Pharmacol	Randomized, single blind	Population: Postmenopausal women with HTN Mean age range (yr): 54–56 Patients (G1 = 45, G2 = 47)	G1: Moexipril^4^	G2: Nitrendipine (0)	15 mg
Akat PB, 2010	Indian J Pharmacol	Randomized, open label	Population: HTN Patients (G1 = 40, G2 = 40)	G1: Enalapril^5^	G2: Telmisartan (0)	10 mg
Amerena A, 2002	Int J Med Res	Randomized, open label	Population: HTN Mean age range (yr): 51–52 Patients (G1 = 255, G2 = 261)	G1: Enalapril^23^	G2: Telmisartan^2^	10 mg
Arashi H, 2020	Am Heart J	Randomized, double blind	Population: Heart transplant Mean age range (yr): 50–54 Patients (G1 = 45, G2 = 46)	G1: Ramipril^20^, *	G2: Placebo (0)	20 mg
Baptista LC, 2019	Clin Med	Randomized, double blind (allocation concealment)	Population: HTN Mean age range (yr): 67–72 Patients (G1 = 10, G2 = 13, G3 = 8)	G1: Perindopril^2^	G2: Losartan (0) G3: HCTZ (0)	4 mg
Benz J, 1997		Randomized, double blind	Population: HTN Mean age range (yr): 52–56 Patients (G1 = 45, G2 = 42, G3 = 43)	G1: Lisinopril^32^, *	G2: Valsartan^9^ G3: HCTZ^8^	10 mg
Bicknell CD, 2016	Eur Heart J	Randomized, single blind	Population: abdominal aortic aneurysms Mean age range (yr): 70–71 Patients (G1 = 73, G2 = 72, G3 = 79)	G1: Perindopril^3^, *	G2: Amlodipine^1^ G3: Placebo (0)	10 mg
Black HR, 1997	J Hum Hypertens	Randomized, open label	Population: Mean age range (yr): 53–54 Patients (G1 = 187, G2 = 364, G3: 183)	G1: Lisinopril^16^	G2: Valsartan (0) G3: Placebo (0)	20 mg
Botero R, 2000	Int J Cardiol	Randomized, open label	Population: HTN Mean age range (yr): 53–57 Patients (G1 = 64, G2 = 64)	G1: Enalapril^7^	G2: Valsartan^2^	20 mg
Breeze E, 2001	J Hum Hypertens	Randomized, double blind	Population: Hypertension Patients: (G1 = 262, G2 = 261)	G1: Enalapril^19^	G2: Eprosartan^8^	
Campo C, 2001	J Clin Hypertens	Randomized, open label, parallel	Population: HTN Mean age range (yr): 43–45 Patients: (G1 = 45, G2 = 40, G3 = 45, G4 = 46)	G1: Lisinopril^5^	G2: Atenolol (0) G3: Nisoldipine (0) G4: Losartan (0)	40 mg
Chan P, 1997	J Clin Pharmacol	Randomized, double blind	Population: Confirmed ACEI cough HTN Mean age range (yr): 72–74 Patients: (G1 = 28, G2 = 28, G3 = 28)	G1: Lisinopril^27^, *	G2: Losartan^6^ G3: Metolazone^5^	10 mg
Chen JH, 2004	J Clin Pract	Randomized, double blind	Population: HTN Mean age range (yr): 49–53 Patients: (G1 = 76, G2 = 71)	G1: Enalapril^3^, *	G2: Telmisartan (0)	10 mg
Cheung BY, 1999	Br J Clin Pharmacol	Randomized, double blind	Population: LVH Mean age range (yr): 44–54 Patients: (G1 = 17, G2 = 16)	G1: Fosinopril^4^	G2: Placebo (0)	20 mg
Chockalingam A, 2004	Am Heart J	Randomized, double blind	Population: Aortic stenosis Mean age range (yr): 43–46 Patients: (G1 = 34, G2 = 18)	G1: Enalapril^4^	G2: Placebo (0)	20 mg
Chrysant SG, 1993	Clin Pharmacol Ther	Randomized, double blind	Population: HTN Mean age range (yr): 51–55 Patients: (G1 = 230, G2 = 59)	G1: Perindopril^29^, *	G2: Placebo^2^	16 mg
Cleland JG, 1995	Brit Heart J	Randomized, double blind	Population: HF Patients: (G1 = 20, G2 = 20)	G1: Enalapril^1^	G2: Placebo (0)	40 mg
Coca A, 2002	Clin Ther	Randomized, double blind	Population: HTN Mean age range (yr): 50–52 Patients: (G1 = 123, G2 = 115)	G1: Enalapril^10^, *	G2: Irbesartan^1^	20 mg
Cohen EP, 2008	Int J Radiation Oncology Biol Phys	Randomized, double blind	Population: BMT nephropathy Mean age range (yr): Patients (G1 = 28, G2 = 27)	G1: Captopril (0)	G2: Placebo^1^	
Cushman WC, 1996	Am J Heart	Randomized, double blind	Population: HTN Mean age range (yr): 52 ‐ 55 Patients: (G1 = 439, G2 = 302, G3 = 150)	G1: Enalapril^12^	G2: Diltiazem SR^4^ G3: Placebo (0)	5 mg
Cuspidi C, 2002	J Hypertens	Randomized, double blind	Population: LVH Patients: (G1 = 105, G2 = 91)	G1: Enalapril^9^	G2: Candesartan^3^	10 mg
Dequattro V, 1997	Clin Exp Hypertens	Randomized, double blind	Population: HTN Mean age range (yr): 55 Patients: (G1 = 267, G2 = 378, G3 = 141)	G1: Trandolapril^19^ G2: Trandolapril + Verapamil^14^	G2: Verapamil^5^	8 mg
Derosa G, 2003	Clin Ther	Randomized, double blind	Population: T2DM + HTN Mean age range (yr): 53–55 Patients: (G1 = 49, G2 = 47)	G1: Perindopril^2^	G2: Candesartan (0)	4 mg
Dickstein K 1995	J Am Coll Pharmacol	Randomized, double blind	Population: HF Mean age range (yr): 52–65 Patients: (G1 = 58, G2 = 108)	G1: Enalapril^4^	G2: Losartan^4^	20 mg
Dickstein K, 2002	Lancet	Randomized, double blind	Population: MI Mean age range (yr): 67 Patients: (G1 = 2733, G2 = 2744)	G1: Captopril^61^	G2: Losartan^47^	45 mg
Dunselman PH, 2001	Int J Cardiol	Randomized, double blind	Population: HF Mean age range (yr): 63–65 Patients: (G1 = 77, G2 = 301)	G1: Enalapril^4^	G2: Telmisartan^9^	20 mg
Eguchi K, 2003	Am J Cardiol	Randomized, double blind	Population: HTN Mean age range (yr): 69 Patients: (G1 = 73, G2 = 73)	G1: Lisinopril^9^	G2: Candesartan^2^	20 mg
Eisner GM, 1991	Am J Heart	Randomized, double blind	Population: HTN Mean age range (yr): 24–74 Patients: (G1 = 82, G2 = 78)	G1: Enalapril^4^	G2: Isradipine (0)	20 mg
Elliott WJ, 1999	J Hum Hypertens	Randomized, double blind	Population: HTN Mean age range (yr): 55–56 Patients: (G1 = 264, G2 = 264)	G1: Enalapril^14^, *	G2: Eprosartan^4^	20 mg
EURopean trial, 2003	Lancet	Randomized, Double blind	Population: CHD Mean age range (yr): 60 Patients: (G1 = 6110, G2 = 6108)	G1: Perindopril^161^, *	G2: Placebo^17^	8 mg
Fan XH, 2008	Ann Pharmacother	Randomized, double blind	Population: HTN Mean age range (yr): 58–59 Patients: (G1 = 976, G2 = 594, G3 = 891, G4 = 947)	G1: Captopril^139^	G2: Atenolol (0) G3 HCTZ (0) G4: Nifedipine SR (0)	50 mg
Fogari R, 2000	Am J Hypertens	Randomized, open label	Population: Microalbuiminuria Mean age range (yr): 61–63 Patients: (G1 = 102, G2 = 103, G3 = 104)	G1: Fosinopril^2^ G2: Fosinopril + Amlodipine^1^	G3: Amlodipine (0)	30 mg
Fogari R, 2005	Eur J Clin Pharmacol	Randomized, open label	Population: Microalbuiminuria Mean age range (yr): 59 ‐ 60 Patients: (G1 = 61, G2 = 60)	G1: Lisinopril^2^	G2: Manidipine (0)	10 mg
Gavras I, 1999	Curr Med Res Opin	Randomized, double blinded	Population: HTN Mean age range (yr): 55–56 Patients: (G1 = 264, G2 = 264)	G1: Enalapril^59^, *	G2: Eprosartan^34^	5 mg
Gradman AH, 1995	Hypertension	Randomized, double blind	Population: HTN Mean age range (yr): 52–56 Patients: (G1 = 83, G2 = 415, G3 = 78)	G1: Enalapril^7^	G2: Losartan^14^ G3: Placebo^2^	20 mg
Gross O, 2020	Kid Int	Randomized, open label	Population: Alport's syndrome Mean age range (yr): 7–9 Patients: (G1 = 53, G2 = 37)	G1: Ramipril^2^	G2: Placebo (0)	6 mg
Gueret P, 1990	Drugs	Randomized, double blind	Population: HTN Mean age range (yr): 55–58 Patients: (G1 = 68, G2 = 68)	G1: Enalapril^4^	G2: Nifedipine (0)	20 mg
Guitard C, 1997	Cardio Drugs Ther	Randomized, double blind	Population: HTN Mean age range (yr): 56–58 Patients (G1 = 100, G2 = 101, G3 = 50)	G1: Spirapril (0) G2: Enalapril^1^	G3: Placebo (0)	G1: 6 mg G2: 20 mg
Hajjar I, 2020	JAMA	Randomized, double blind	Population: HTN Mean age range (yr): 65–66 Patients (G1 = 89, G2 = 87)	G1: Lisinopril^24^, *	G2: Candesartan^7^	
Halimi JM, 2007	Clin transplant	Randomized, open label	Population: Renal transplant Mean age range (yr): 35–36 Patients (G1 = 70, G2 = 70, G3 = 58)	G1: Enalapril^11^ G2: Enalapril + amlodipine^6^	G2: Amlodipine (0)	20 mg
Hart W, 1993	Postgrad Med J	Randomized, double blind	Population: HTN Mean age range (yr): 52–56 Patients: (G1 = 63, G2 = 64)	G1: Lisinopril^8^, *	G2: Nifedipine (0)	40 mg
Himmelmann A, 2001	Blood Press	Randomized, double blind	Population: HTN Mean age range (yr): 54–55 Patients: (G1 = 194, G2 = 196)	G1: Enalapril^15^, *	G2: Candesartan^7^	20 mg
HOPE, 2000	NEJM	Randomized, double blind	Population: High risk CAD Mean age range (yr): 66 Patients: (G1 = 4645, G2 = 5652)	G1: Ramipril (340)*	G2: Placebo^85^	10 mg
Hou FF, 2006	NEJM	Randomized, double blind	Population: CRI Mean age range (yr): 44–45 Patients: (G1 = 112, G2 = 112)	G1: Benazepril^1^	G2: Placebo (0)	20 mg
Hou FF, 2007	J Am Soc Nephrol	Randomized, open label	Population: Proteinuria or CRI Mean age range (yr): 49–51 Patients: (G1 = 180, G2 = 180)	G1: Benazepril^32^	G2: Losartan (0)	40 mg
Ishimitsu T, 2007	Nephr	Randomized, double blind	Population: CRI Mean age range (yr): 53 Patients: (G1 = 15, G2 = 15)	G1: Benazepril^2^	G2: Placebo (0)	5 mg
Johnson BF, 1995	Hypertens	Randomized, double blind	Population: HTN Mean age range (yr): 54 Patients: (G1 = 82, G2 = 78)	G1: Enalapril^12^	G2: Isradipine^6^	40 mg
Juarez GF, 2013	Am J Kidney Dis	Randomized, double blind, allocation concealment	Population: diabetic nephropathy Mean age range (yr): 63–68 Patients: (G1 = 35, G2 = 28, G3 = 70)	G1: Lisinopril^1^, * G2: Lisinopril + irbesartan^4^	G3: Irbesartan (0)	40 mg
Karlberg BE, 1999	J Hypertens	Randomized, double blind	Population: HTN Mean age range (yr): 57–61 Patients: (G1 = 139, G2 = 139)	G1: Enalapril^22^	G2: Telmisartan^9^	20 mg
Katoch N, 2019	Asian J Pharm Clin Res	Randomized, open label	Population: MI Mean age range (yr): 55–56 Patients: (G1 = 50, G2 = 50)	G1: Ramipril^3^	G2: Losartan (0)	2.5 mg
Ke YS, 2003	Acta Pharmacol	Randomized, open label	Population: HTN Mean age range (yr): 48–50 Patients: (G1 = 30, G2 = 30, G3 = 30)	G1: Benazepril^3^ G2: Benazepril + valsartan^2^	G3: Valsartan (0)	10 mg
Kereiakes DJ, 2007	Am J Cardiovasc	Randomized, double blind	Population: HTN Mean age range (yr): 54–56 Patients: (G1 = 96, G2 = 94)	G1: Benazepril^11^	G2: Olmesartan^2^	20 mg
Kitzman DW, 2010	Circ Heart Fail	Randomized, double blind	Population: HF Mean age range (yr): 69 ‐ 70 Patients: (G1 = 35, G2 = 36)	G1: Enalapril^1^	G2: Placebo (0)	20 mg
Ko GT, 2005	Adv Ther	Randomized, double blind	Population: T2DM with albuminuria Mean age range (yr): 59–62 Patients: (G1 = 20, G2 = 22)	G1: Enalapril^7^	G2: Valsartan (0)	10 mg
Kober LK 1995	N Engl J Med	Randomized, double blind	Population: LVD Mean age range (yr): 67 Patients: (G1 = 876, G2 = 873)	G1: Trandolapril^39^, *	G2: Placebo^13^	2 mg
Koch B, 1999	J Hum Hypertens	Randomized, double blind	Population: Post‐menopausal Mean age range (yr): 56–57 Patients: (G1 = 47, G2 = 48)	G1: Moexipril^6^	G2: Placebo (0)	15 mg
Kroll GA, 2016	Lancet	Randomized, double blind, allocation concealment	Population: Renal transplant Mean age range (yr): 52–54 Patients: (G1 = 104, G2 = 109)	G1: Ramipril^4^	G2: Placebo (0)	10 mg
Lacourciere Y, 2000	Clin Ther	Randomized, double blind	Population: HTN Mean age range (yr): 70–71 Patients: (G1 = 71, G2 = 70)	G1: Enalapril^11^, *	G2: Irbesartan^3^	20 mg
Lacourciere Y, 2006	Am J Hypertens	Randomized, open label, blinded end point	Population: HTN Mean age range (yr): 52 Patients: (G1 = 407, G2 = 405)	G1: Ramipril^33^	G2: Telmisartan^1^	10 mg
Larochelle P, 1997	Am J Cardiol	Randomized, double blind	Population: HTN Mean age range (yr): 52–53 Patients: (G1 = 61, G2 = 121)	G1: Enalapril^8^	G2: Irbesartan^3^	20 mg
Leonetti G, 2006	Blood Press	Randomized, double blind	Population: HTN Mean age range (yr): 51 Patients: (G1 = 114, G2 = 122)	G1: Zofenopril^2^	G2: Candesartan (0)	30 mg
Leu HB, 2004	Jpn Heart J	Randomized, double blind	Population: HTN Mean age range (yr): 57–59 Patients: (G1 = 20, G2 = 22)	G1: Enalapril^5^	G2: Eprosartan^3^	20 mg
Lohmann FW, 1999	Clin Drug Invest	Randomized, open label	Population: HTN Mean age range (yr): 67 Patients: (G1 = 293, G2 = 439, G3 = 309)	G1: Ramipril^6^	G2: Felodipine (0) G3: ISMN^9^	5 mg
Lonn EM, 2009	J Am Coll Cardiol	Randomized, double blind	Population: IGT or IFG Mean age range (yr): 53–54 Patients: (G1 = 715, G2 = 710)	G1: Ramipril^53^, *	G2: Placebo^11^	15 mg
MacGregor MS, 2005	Nephron Clin Pract	Randomized, open label	Population: renal failure Mean age range (yr): 50 Patients: (G1 = 553, G2 = 549)	G1: Ramipril^13^	G2: Olmesartan^2^	10 mg
Malacco E, 2010	J Hypertens	Randomized, double blind	Population: HTN Mean age range (yr): 72 Patients: (G1 = 213, G2 = 222)	G1: Zofinopril + HCTZ^6^, *	G2: Irbesartan + HCTZ (0)	10 mg
Malacco E, 2004	Clin Ther	Randomized, double blind	Population: HTN Mean age range (yr): 54 Patients: (G1 = 609, G2 = 604)	G1: Lisinopril^44^	G2: Valsartan^6^	20 mg
Mallion JM, 2011	Am J Hypertens	Randomized, double blind	Population: HTN Mean age range (yr): 71–72 Patients: (G1 = 175, G2 = 170)	G1: Ramipril^4^	G2: Olmesartan (0)	10 mg
Malmqvist K, 2000	J Hypertens	Randomized, double blind	Population: HTN women Mean age range (yr): 57–58 Patients: (G1 = 146, G2 = 140, G3 = 143)	G1: Enalapril^19^	G2: Candesartan (0) G3: HCTZ^6^	20 mg
Marketou ME, 2008	J Hum Hypertens	Randomized, open label	Population: DM normotensive Mean age range (yr): 63–64 Patients: (G1 = 32, G2 = 30)	G1: Perindopril^2^	G2: Placebo (0)	4 mg
Mauer M, 2009	N Engl J Med	Randomized, double blind	Population: T1DM Mean age range (yr): Patients: (G1 = 94, G2 = 96, G3 = 95)	G1: Enalapril^12^	G2: Losartan^6^ G3: Placebo^4^	20 mg
Menne J, 2008	J Hypertens	Randomized, double blind	Population: HTN with microalbuminuria Mean age range (yr): 57–59 Patients: (G1 = 47, G2 = 40, G3 = 42)	G1: Lisinopril^2^ G2: Lisinopril/ valsartan^1^	G3: Valsartan (0)	40 mg
Messerli F, 1998	Am J Hypertens	Randomized, double blind	Population: HTN Mean age range (yr): Patients: (G1 = 159, G2 = 163, G3 = 152, G4 = 157)	G1: Trandolapril^12^ G2: Trandolapril/ verapamil^9^	G3: Placebo^4^ G4: Verapamil^1^	4 mg
Mimran A, 1998	J Hum Hypertens	Randomized, double blind	Population: HTN Mean age range (yr): 58 Patients: (G1 = 102, G2 = 98)	G1: Enalapril^15^, *	G2: Irbesartan^7^	40 mg
Morgan TO, 1992	Am J Hypertens	Randomized, double blind	Population: HTN Mean age range (yr): 67 Patients: (G1 = 10, G2 = 310, G3 = 10)	G1: Enalapril^1^ G2: Enalapril + felodipine^2^	G3: Felodipine^2^	10 mg
Nakamura T, 2009	Int Heart J	Randomized, double blind	Population: HTN Mean age range (yr): 63–66 Patients: (G1 = 27, G2 = 26)	G1: Perindopril^2^	G2: Telmisartan (0)	8 mg
Nalbantgil I, 2004	Int J Clin Pract	Randomized, double blind	Population: HTN Mean age range (yr): 50 ‐ 51 Patients: (G1 = 30, G2 = 30)	G1: Perindopril^2^	G2: Telmisartan (0)	4 mg
Neutel JM, 1999	Am J Ther	Randomized, double blind	Population: HTN Mean age range (yr): 53 Patients: (G1 = 193, G2 = 385)	G1: Lisinopril^7^, *	G2: Telmisartan^3^	40 mg
Niseen SE, 2004	JAMA	Randomized, double blind, allocation concealment	Population: CAD Mean age range (yr): 57–58 Patients: (G1 = 673, G2 = 663, G3 = 655)	G1: Enalapril^84^	G2: Amlodipine^34^ G3: Placebo^38^	20 mg
Northridge DB, 1993	Eur Heart J	Randomized, double blind	Population: HF Mean age range (yr): 57–62 Patients: (G1 = 60, G2 = 30)	G1: Quinapril^6^	G2: Placebo^2^	20 mg
Omvik P, 1994	Br J Clin Pract	Randomized, double blind	Population: HTN Mean age range (yr): 54 Patients: (G1 = 230, G2 = 231)	G1: Enalapril^29^	G2: Amlodipine^9^	40 mg
ONTARGET, 2008	NEJM	Randomized, double blind	Population: Vascular disease Mean age range (yr): 66 Patients: (G1 = 8576, G2 = 8502, G3 = 8542)	G1: Ramipril (360) G2: Ramipril + telmisartan (392)	G3: Telmisartan^93^	10 mg
Ormesher L, 2020	Hypertens	Randomized, double blind	Population: Preeclampsia Mean age range (yr): 30 ‐ 34 Patients: (G1 = 30, G2 = 30)	G1: Ramipril^3^	G2: Placebo (0)	20 mg
Ostergren J, 1996	Am J Hypertens	Randomized, double blind	Population: HTN Patients: (G1 = 119, G2 = 129)	G1: Enalapril^30^, *	G2: Placebo (0)	40 mg
Otero ML, 2005	Clin Ther	Randomized, double blind	Population: T2DM with HTN Mean age range (yr): 60 ‐ 64 Patients: (G1 = 58, G2 = 53)	G1: Enalapril^6^, *	G2: Manidipine (0)	10 mg
Perico N, 1998	Clin Drug Invest	Randomized, double blind	Population: HTN + renal insufficiency Mean age range (yr): 42–55 Patients: (G1 = 94, G2 = 94)	G1: Lisinopril^1^	G2: Valsartan (0)	10 mg
Pfeffer MA, 2003	NEJM	Randomized, double blind, allocation concealment	Population: LVD Mean age range (yr): 64–65 Patients: (G1 = 4909, G2 = 4885, G3 = 4909)	G1: Captopril (245)* G2: Captopril + valsartan (225)	G3: Valsartan^85^	150 mg
Phakdeekitcharoen P, 2004	Am J Kidney Dis	Randomized, open label	Population: CAPD Mean age range (yr): 48–58 Patients: (G1 = 29, G2 = 29)	G1: Enalapril^7^	G2: Candesartan (0)	10 mg
Philipp T, 1997	BMJ	Randomized, double blind	Population: HTN Mean age range (yr): 53 Patients: (G1 = 220, G2 = 215, G3 = 218, G4 = 218)	G1: Enalapril^6^, *	G2: Atenolol (0) G3: Nitrendipine (0) G4: HCTZ (0)	20 mg
Pitt B, 1997	Lancet	Randomized, double blind	Population: HF Mean age range (yr): 73–74 Patients: (G1 = 370, G2 = 352)	G1: Captopril^14^, *	G2: Losartan (0)	150 mg
Pitt B, 2001	Am J Cardiol	Randomized, open label	Population: Ischemic heart disease Mean age range (yr): 58 Patients: (G1 = 878, G2 = 872)	G1: Quinapril^33^, *	G2: Placebo^2^	20 mg
Prisant LM, 1995	Am Heart J	Randomized, double blind	Population: HTN Mean age range (yr): 53–55 Patients: (G1 = 71, G2 = 72, G3 = 75)	G1: Enalapril^3^	G2: Amlodipine^4^ G3: Bisoprolol + HCTZ (0)	20 mg
Prisant LM, 1998	Am J Ther	Randomized, double blind	Population: Patients: (G1 = 84, G2 = 82, G3 = 78, G4 = 74)	G1: Enalapril^7^	G2: Amlodipine^3^ G3: Bisoprolol (0) G4: Placebo^3^	40 mg
PROGRESS 2001	Lancet	Randomized, open label	Population: Stroke or TIA Mean age range (yr): 63–65 Patients: (G1 = 3051, G2 = 3054)	G1: Perindopril^47^	G2: Placebo^69^	4 mg
Radman S, 2007	Eur J Clin Pharmacol	Randomized, double blind	Population: T2DM or IGT Mean age range (yr): 46–48 Patients: (G1 = 11, G2 = 10, G3 = 10)	G1: Ramipril^3^	G2: Rosiglitazone (0) G3: Placebo (0)	10 mg
Ragot S, 2002	J Human Hypertens	Randomized, open label	Population: HTN Mean age range (yr): 55 Patients: (G1 = 218, G2 = 217)	G1: Perindopril^12^	G2: Telmisartan^2^	4 mg
Ramsey LE, 1995	J Hypertens	Randomized, double blind	Population: HTN Patients: (G1 = 46, G2 = 48, G3 = 41)	G1: Lisinopril^33^	G2: Losartan^14^ G3: HCTZ^14^	20 mg
Reyes‐Marin, FA, 2012	Rev Invest Clin	Randomized, double blind	Population: Peritoneal dialysis Mean age range (yr): 42–49 Patients: (G1 = 30, G2 = 30)	G1: Enalapril^2^	G2: Valsartan^3^	10 mg
Rogstad B, 1994	Eur J Pharmacol	Randomized, double blind	Population: HTN Mean age range (yr): 49 ‐ 51 Patients: (G1 = 49, G2 = 53)	G1: Lisinopril^9^	G2: Nifedipine^2^	10 mg
Rosei EG, 2005	Am J Hypertens	Randomized, double blind	Population: HTN Mean age range (yr): 53–54 Patients: (G1 = 133, G2 = 134)	G1: Enalapril^60^	G2: Nifedipine (0)	20 mg
Rouleau JL, 2008	Circulation	Randomized, double blind, Allocation concealment	Population: CABG Mean age range (yr): 61 Patients: (G1 = 1280, G2 = 1273)	G1: Quinapril (269)	G2: Placebo^140^	20 mg
Ruddy TD, 1997	Cardiovasc Drugs Ther	Randomized, double blind	Population: HTN Mean age range (yr): 51–53 Patients: (G1 = 140, G2 = 138)	G1: Lisinopril^15^	G2: Nisoldipine^6^	20 mg
Ruilope L, 2001	Blood Pressure	Randomized, double blind	Population: HTN Mean age range (yr): 73 Patients: (G1 = 163, G2 = 171)	G1: Enalapril^10^	G2: Eposartan^1^	20 mg
Sabharwal NK, 2005	Clin Drug Invest	Randomized, double blind	Population: HTN Mean age range (yr): 52–54 Patients: (G1 = 43, G2 = 43)	G1: Imidapril^1^	G2: Nifedipine (0)	10 mg
Sampaio RO, 2005	Am J Cardiol	Randomized, open label, blinded outcome, Allocation concealment	Population: Mitral valve prolapse, rheumatic heart disease Mean age range (yr): 38–40 Patients: (G1 = 26, G2 = 21)	G1: Enalapril^1^	G2: Placebo (0)	40 mg
Schaefer F, 2011	J Hypertens	Randomized, double blind	Population: HTN Mean age range (yr): 13 Patients: (G1 = 149, G2 = 151)	G1: Enalapril^10^	G2: Valsartan^9^	20 mg
Schrader H, 2001	BMJ	Randomized, double blind	Population: Migraine Mean age range (yr): 41 Patients: (G1 = 60, G2 = 60)	G1: Lisinopril^8^	G2: Placebo^3^	20 mg
Sega R, 1999	Am J Hypertens	Randomized, double blind	Population: HTN Patients: (G1 = 59, G2 = 59)	G1: Enalapril^2^	G2: Eprosartan^2^	40 mg
Shionoiri I, 1999	J Clin Pharmacol	Randomized, open label	Population: HTN Mean age range (yr): 53 Patients: (G1 = 29, G2 = 31)	G1: Imidapril^28^	G2: Amlodipine^2^	5 mg
Silagy C, 1992	Am J Cardiol	Randomized, double blind	Population: HTN Mean age range (yr): 72 Patients: (G1 = 24, G2 = 23, G3 = 20, G4 = 23)	G1: Enalapril^5^	G2: HCTZ (0) G3: Atenolol^1^ G4: Isradipine (0)	10 mg
SOLVD, 1991	N Engl J Med	Randomized, double blind	Population: HF Mean age range (yr): 60–61 Patients: (G1 = 1285, G2 = 1284)	G1: Enalapril (475)	G2: Placebo (398)	20 mg
Sonbolestan S, 2013	Int J Prev Med	Randomized, double blind	Population: Migraine Mean age range (yr): 31–37 Patients: (G1 = 21, G2 = 19)	G1: Enalapril^3^	G2: Placebo (0)	10 mg
Song J, 2006	Nephrol Dial Transplant	Randomized, double blind	Population: T2DM with kidney disease Mean age range (yr): 49 Patients: (G1 = 8, G2 = 8, G3 = 9)	G1: Ramipril (0) G2: Ramipril + candesartan (0)	G3: Candesartan (0)	10 mg
Spiner J, 2000	Eur J Heart Fail	Randomized, single blind, double blind outcome	Population: MI Mean age range (yr): 65 Patients: (G1 = 101, G2 = 100)	G1: Captopril^26^	G2: Losartan^12^	75 mg
Sumukadas D, 2018	Age and Aging	Randomized, double blind	Population: Postural instability elerly Mean age range (yr): 78 Patients: (G1 = 40, G2 = 40)	G1: Perindopril^4^, *	G2: Placebo (0)	4 mg
Tan F, 2010	Singapore Med J	Randomized, open label	Population: T2DM nephropathy Mean age range (yr): 57–58 Patients: (G1 = 16, G2 = 18)	G1: Enalapril^4^, *	G2: Losartan (0)	20 mg
Tanser P, 2000	Am J Hypertens	Randomized, double blind	Population: HTN Mean age range (yr): 60–61 Patients: (G1 = 66, G2 = 62, G3: 26)	G1: Enalapril^20^	G2: Candesartan^10^ G3: Placebo^3^	10 mg
Tikkamen I, 1995	J Hypertens	Randomized, double blind	Population: HTN Mean age range (yr): Patients: (G1 = 71, G2 = 80)	G1: Enalapril^9^	G2: Losartan^1^	20 mg
Tomlinson B, 1994	Am J Hypertens	Randomized, double blind	Population: HTN Mean age range (yr): 75–76 Patients: (G1 = 16, G2 = 18)	G1: Spirapril^3^	G2: Isradipine^7^	5 mg
Tomlinson B, 2004	Clin Ther	Randomized, double blind	Population: HTN Mean age range (yr): 59–61 Patients: (G1 = 40, G2 = 40)	G1: Enalapril^13^, *	G2: Amlodipine^3^	20 mg
Toto R, 1996	Am J Kidney Dis	Randomized double blind	Population: normotensive with proteinuria Patients: (G1 = 15, G2 = 15)	G1: Ramipril^1^	G2: Placebo (0)	5 mg
Townsend R, 1995	Clin Ther	Randomized, double blind	Population: HTN Mean age range (yr): 54 ‐ 55 Patients: (G1 = 136, G2 = 132)	G1: Enalapril^13^	G2: Losartan^5^	10 mg
Van Der Does R, 2001	J Int Med Res	Randomized, double blind	Population: HTN Mean age range (yr): 54 Patients: (G1 = 157, G2 = 162)	G1: Imidapril^9^	G2: Nifedipine (0)	10 mg
Velasco M, 1991	J Cardiovasc Pharmacol	Randomized, double blind	Population: HTN Mean age range (yr): 50–53 Patients: (G1 = 19, G2 = 21)	G1: Captopril^2^	G2: Amlodipine^1^	100 mg
Verkaaik R, 1991	J Cardiovasc Pharmacol	Randomized, double blind	Population: HTN Mean age range (yr): 53 Patients: (G1 = 44, G2 = 44)	G1: Enalapril^3^	G2: Nitredipine (0)	20 mg
Weber M, 2012	J Clin Hypertens	Randomized, double blind	Population: HTN Mean age range (yr): 47–50 Patients: (G1 = 189, G2 = 189, G3 = 188, G4 = 95)	G1: Lisinopril^6^ G2: Lisinopril + Nebivolol^3^	G3: Nebivolol^4^ G4: Placebo^1^	40 mg
Wei F, 2011	Heart	Randomized, open label, blinded outcomes	Population: HTN Mean age range (yr): 57–58 Patients: (G1 = 255, G2 = 257)	G1: Imidapril^8^	G2: Candesartan (0)	5 mg
White M, 2002	Am Heart J	Randomized, double blind	Population: HTN Mean age range (yr): 54–56 Patients: (G1 = 99, G2 = 103, G3 = 109, G4 = 46)	G1: Enalapril^12^	G2: Losartan^11^ G3: Verapamil^11^ G4: Placebo (0)	20 mg
White M, 2004	Am Heart J	Randomized, double blind	Population: HTN Mean age range (yr): 53–55 Patients: (G1 = 131, G2 = 128)	G1: Ramipril^10^	G2: Diltiazem^1^	20 mg
Widimsky J, 1995	Eur J Clin Pharmacol	Randomized, double blind	Population: HF Mean age range (yr): 57 Patients: (G1 = 152, G2 = 48, G3 = 48)	G1: Spirapril^1^ G2: Enalapril^3^	G3: Placebo (0)	6 mg 10 mg
Williams B, 2006	J Hypertens	Randomized, open label, blinded outcomes	Population: HTN Mean age range (yr): 53 Patients: (G1 = 404, G2 = 397)	G1: Ramipril^23^	G2: Talmisartan^2^	5 mg
Wu S, 2004	Blood Vessels	Randomized, open label	Population: HTN Mean age range (yr): 63–66 Patients: (G1 = 41, G2 = 40, G3 = 40)	G1: Lisinopril^13^, *	G2: Amlodipine (0) G3: Losartan^1^	10 mg
Yokota T, 2010	Heart Vessels	Randomized, open label	Population: MI Mean age range (yr): 64–66 Patients: (G1 = 81, G2 = 82)	G1: Enalapril^2^, *	G2: Telmisartan (0)	10 mg
Zannad F, 1999	J Hypertens	Randomized, double blind	Population: HTN Mean age range (yr): Patients: (G1 = 49, G2 = 47)	G1: Perindopril^7^	G2: Amlodipine^1^	8 mg
Zi M, 2003	Cardiovasc Drugs Ther	Randomized, double blind	Population: HF Mean age range (yr): 77–78 Patients: (G1 = 36, G2 = 38)	G1: Quinapril^6^	G2: Placebo^1^	40 mg

Abbreviations: BMT, bone marrow transplant; CAD, coronary artery disease; CABG, coronary artery bypass grafting; CRI, chronic renal insufficiency; HCTZ, hydrochlorothiazide; HF, heart failure; HTN, hypertension; IGT, impaired glucose tolerance; LVD, left ventricular dysfunction; LVH, Left ventricular hypertrophy; T1DM, type 1 diabetes mellitus; T2DM, type 2 diabetes mellitus; TIA, transient ischemic attack.

* Patients required discontinuation due to cough.

**FIGURE 2:**
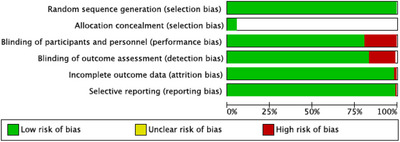
Bias risk assessment for the studies.

The quality of eligibility studies is shown in Figure 2 and the overall quality has low risk of bias. Only four studies disclosed the allocation of concealment.

### 3.1 Network meta‐analysis

**FIGURE 3:**
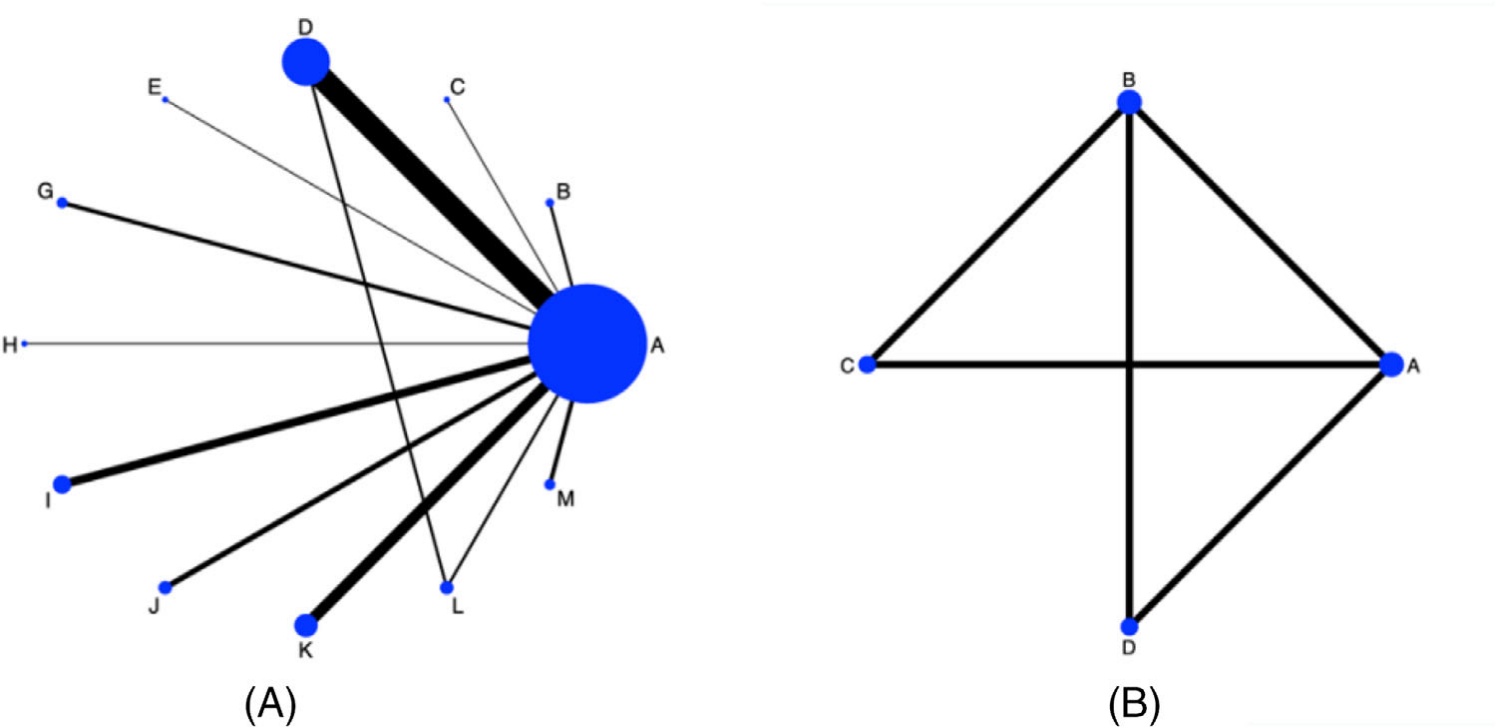
Network map. A: The network map of single ACEIs comparison for cough risk ratios. The width of the black line is positively proportional to the number of trials including every pair of treatments, whereas every circle size is positively proportional to the total number of participants for each treatment. A: Placebo; B: Benazepril; C: Captopril; D: Enalapril; E: Fosinopril; G: Lisinopril; H: Moexipril; I: perindopril; J: Quinapril; K: Ramipril; L: Spirapril; M: Trandolapril. Network map B: The network map of different groups comparisons for cough risk ratios. A: Placebo; B: ACEIs group; C: ARBs group; D: CCBs group.

The network map illustrates the comparison of eleven different ACEIs based on the indirect evaluation of cough risk ratios (Figure 3A). The comparisons between the ACEIs group, ARBs group, CCBs group, and placebo are illustrated in the network map (Figure 3B) based on the combination data of cough risk ratios.

#### 3.1.1 Network meta‐analysis for cough induced by different ACEIs

**FIGURE 4:**
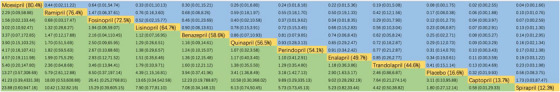
Single ACEI interventions network meta‐analysis for cough. League table showing results of the network meta‐analysis comparing cough of all treatments including RR and 95% credible intervals. RR > 1 means the top‐left treatment is better. The league table represents the relative risk with 95% confidence interval of single ACEIs compared with placebo. The probabilities beside the ACEIs are the treatment ranking based on SUCRA from left to right. The treatment drugs divide the figure into upper (blue colored) and lower (green colored) sections. For the lower section, the efficacy estimate is the ratio of the column defining treatment to the row defining treatment. For the upper part, the efficacy estimate was the ratio of the row defining treatment to the column defining treatment. The lower and the upper portions’ results are mutually reciprocal. The relative risk ratio in each treatment is compared to the treatment to the right in the same row.

The direct and indirect cough development comparisons of different single ACEIs were combined to perform the network meta‐analysis process. Based on the direct and indirect evidence extracted from the included RCTs, the comparisons between each ACEI and alternative ACEI or placebo were completed in the network forest. The ranking order from the maximal to the minimal cough risk was performed and demonstrated by the surface under the cumulative ranking curves (SUCRA). For each treatment, the ranking indicates which of the ACEI is more likely to cause cough and which one is less likely to cause cough. In Figure 4, moexipril ranked as number one for inducing cough (SUCRA 80.4%). The order for the rest of the ACEIs are as follows: ramipril (SUCRA 76.4%), fosinopril (SUCRA 72.5%), lisinopril (SUCRA 64.7%), benazepril (SUCRA 58.6%), quinapril (SUCRA 56.5%), perindopril (SUCRA 54.1%), enalapril (SUCRA 49.7%), trandolapril (SUCRA 44.6%), captopril (SUCRA 13.7%), and spirapril (SUCRA 12.3%) (Table 2).

**TABLE 2:** Ranking of ACEI induced cough compared to placebo based on SUCRA.

ACE inhibitor	SUCRA value
Ramipril	76.4%
Fosinopril	72.5%
Lisinopril	64.7%
Benazepril	58.6%
Quinapril	56.5%
Perindopril	54.1%
Enalapril	49.7%
Trandolapril	44.6%
Captopril	13.7%
Spirapril	12.3%

With the exceptions of spirapril and captopril, other ACEIs resulted in higher risk rations (RRs) of cough compared with placebo. Spirapril ranked the least and captopril ranked next least probability for cough, but no statistical significance was observed (spirapril vs. placebo: RR = 1.8, 95% CI: 0.27–12.14; captopril vs. placebo: RR = 3.11, 95% CI: 0.10‐95.88). Ramipril ranked the second highest risk with RR = 5.79 (95% CI: 2.61–12.88) times risk of cough compared with placebo and 10.42 times risk compared with spirapril (95% CI: 1.32–82.16). Lisinopril has 4.39 times risk of cough compared with placebo (95% CI: 1.15‐16.81). Quinapril has 3.41 times of risk compared with placebo (95% CI: 1.36‐8.49). Perindopril and enalapril, two commonly used ACEIs, had 3.18, times and 2.9 times risk of developing cough respectively, and the RRs are statistically significant (perindopril vs. placebo: 95% CI: 1.42–7.13, enalapril vs. placebo: 95% CI: 1.63–5.17). Moexipril, fosinopril, benazepril, and trandolapril have higher risk of inducing cough compared with placebo, but no statistical significance was observed (Figure 4). The 95% CI of the inconsistency factors of the existing closed‐loops did not exclude zero implying that there was no observed inconsistency between direct and indirect evidence.

### 3.2 The different treatment comparisons for cough risk ratios

**FIGURE 5:**
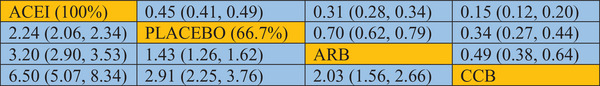
Five different types of anti‐hypertension drugs network meta‐analysis for cough. League table showing results of the network meta‐analysis comparing cough of five types of drugs including RR and 95% credible intervals. RR > 1 means the top‐left treatment is better. The league table represents the relative risk with 95% confidence interval of single ACEIs compared with placebo. The probabilities beside the ACEIs are the treatment ranking based on SUCRA from left to right. The treatment drugs divide the figure into upper (blue colored) and lower (green colored) sections. For the lower section, the efficacy estimate is the ratio of the column defining treatment to the row defining treatment. For the upper part, the efficacy estimate was the ratio of the row defining treatment to the column defining treatment. The lower and the upper portions’ results are mutually reciprocal. The relative risk ratio in each treatment is compared to the treatment to the right in the same row.

**FIGURE 6:**
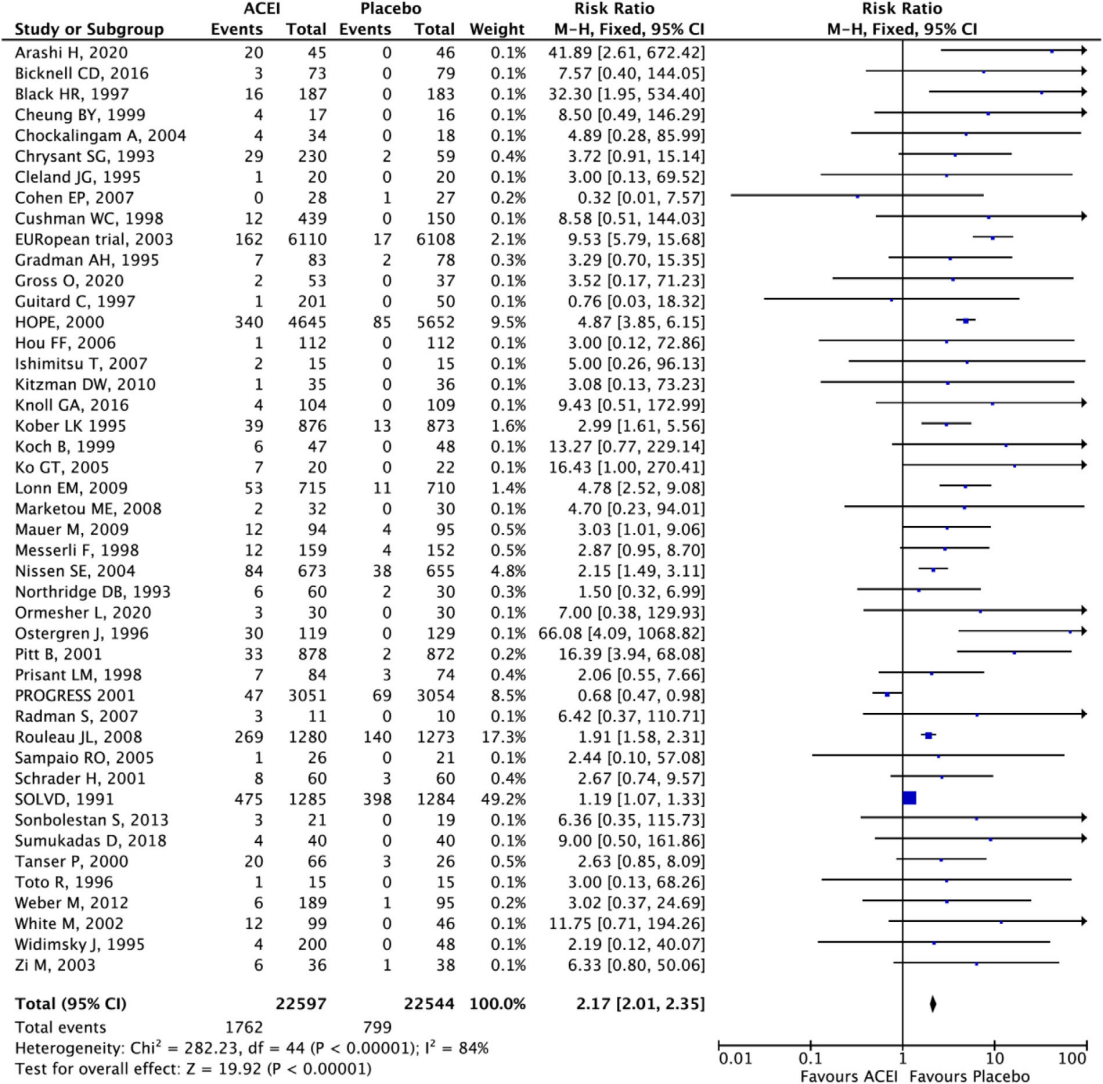
Forest plot comparing ACEI vs. placebo.

**FIGURE 7:**
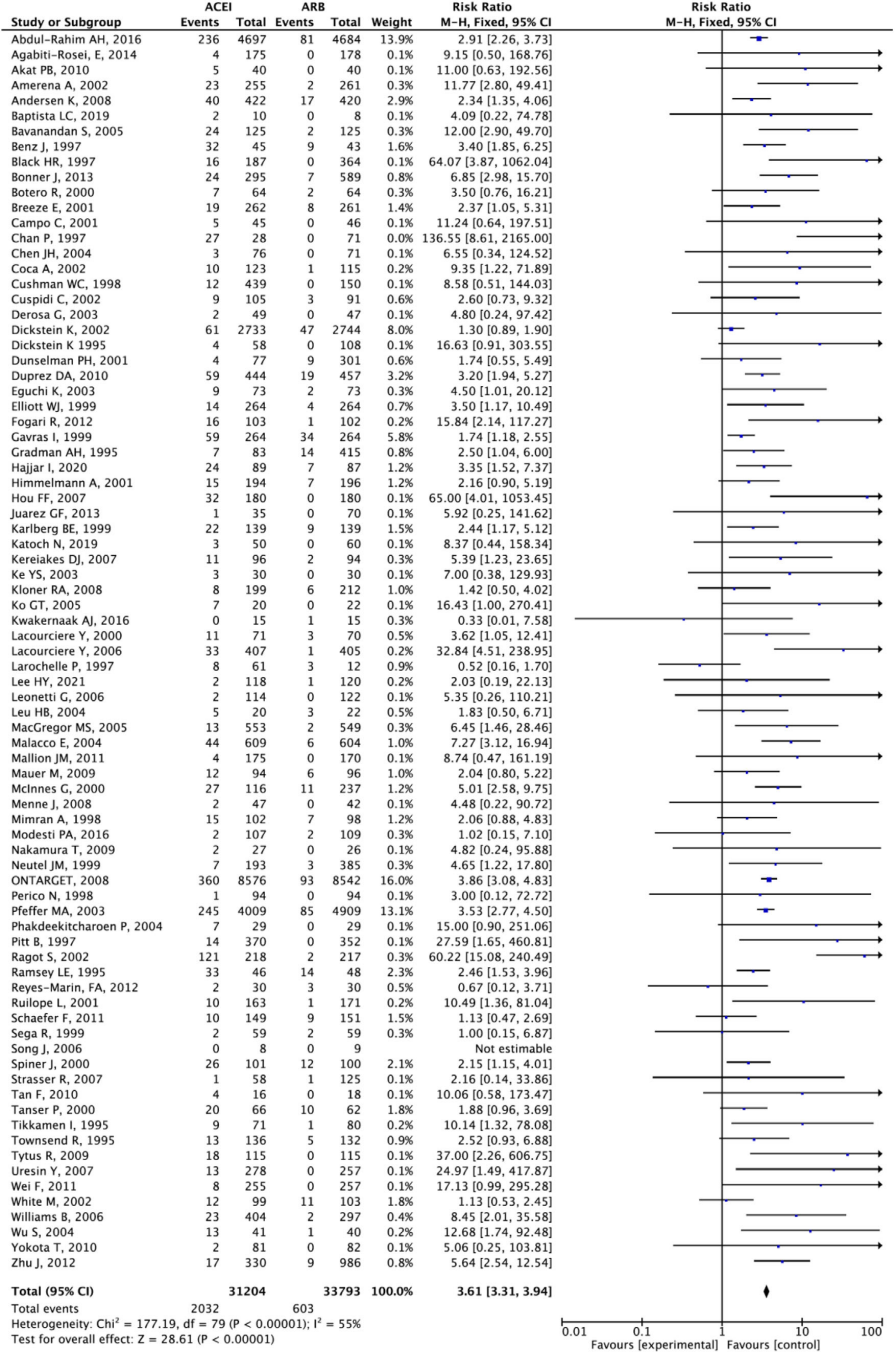
Forest plot comparing ACEI vs. ARB.

**FIGURE 8:**
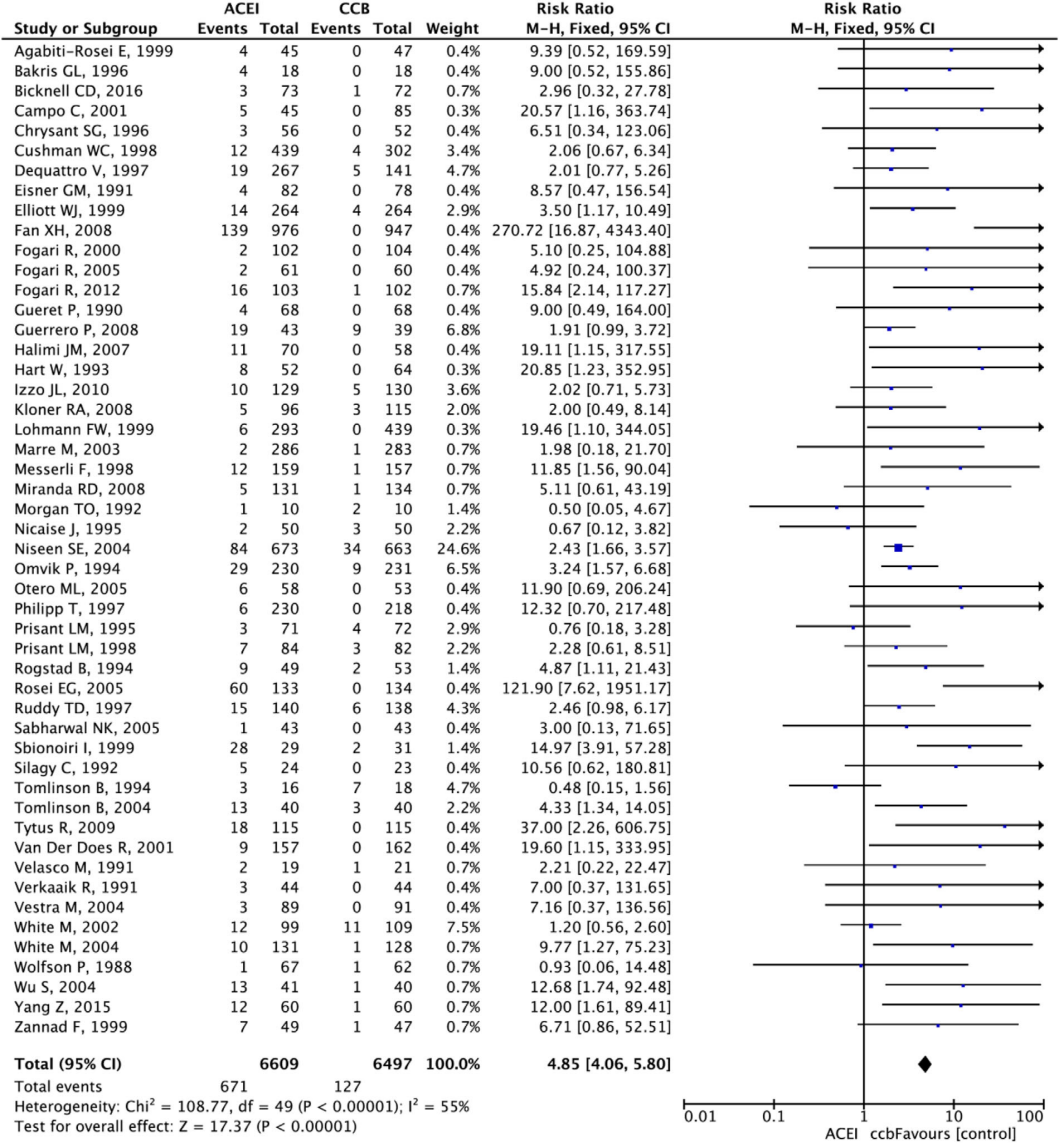
Forest plot comparing ACEI vs. CCB.

After applying the combination data of cough events from different classes of anti‐hypertension drugs, the risk ratios between ACEIs group, ARBs group, CCBs group, Sacubitril/valsartan and placebo are statistically significant with narrow confidence intervals. In Figure 5, the ACEI group ranked the top among five groups based on the SUCRA (99.9%). The next order was placebo (SUCRA, 50.7%), ARBs (SUCRA, 25%), and the CCBs ranked the least risk of inducing cough (SUCRA, 0%). ACEI have 2.24 times the risk of developing cough compared with placebo (95% CI: 2.06–2.3), 3.2 times compared with ARBs (95% CI: 2.9‐3.53), and 6.5 times compared with CCBs (95% CI: 5.07‐8.34). ARBs have 2.03 times the cough risk ratios compared with CCBs (95% CI: 1.56–2.66). Forest plots for the comparisons are presented in Figures 6, 7, 8 respectively. All comparisons were statistically significant. The 95% CI of the inconsistency factors of the existing closed‐loops did not exclude zero implying that there was no inconsistency observed between direct and indirect evidence.

### 3.3 Publication bias

**FIGURE 9:**
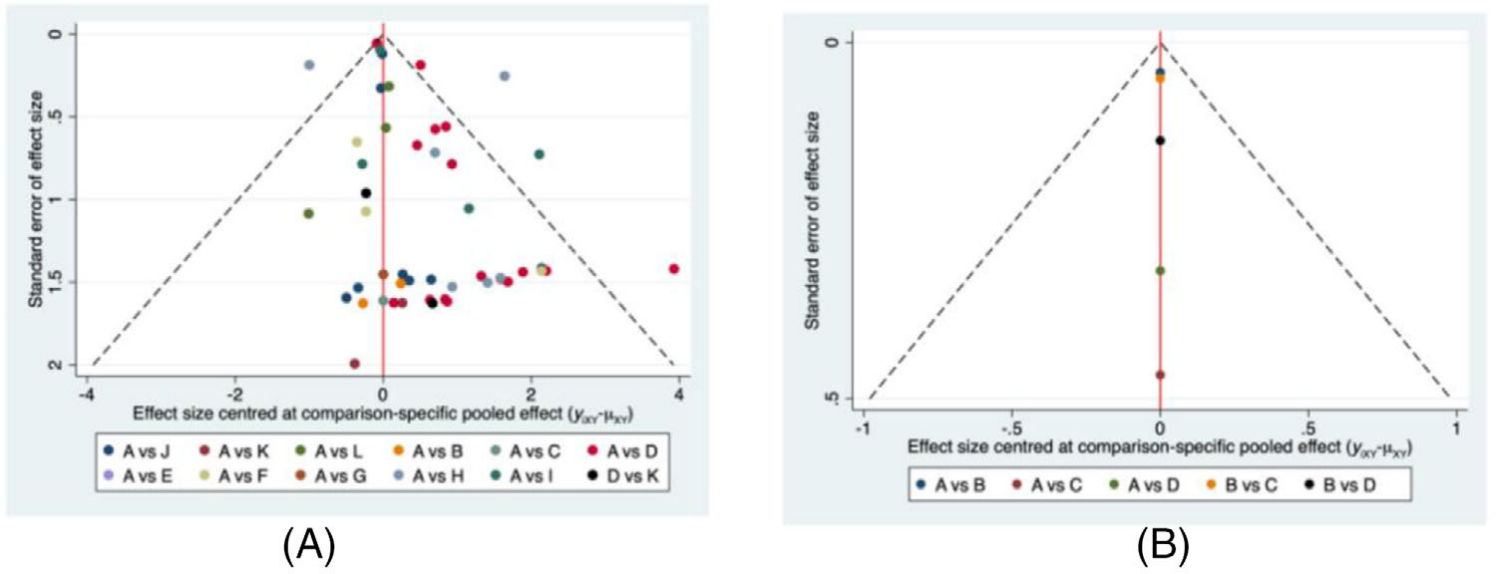
Comparison‐adjusted funnel plots of cough development. A: single ACEI versus placebo network meta‐analysis. A: Placebo; B: Benazepril; C: Captopril; D: Enalapril; E: Fosinopril; G: Lisinopril; H: Moexipril; I: Perindopril; J: Quinapril; K: Ramipril; L: Spirapril; M: Trandolapril. B: the five groups of anti‐hypertension drugs comparisons network meta‐analysis. A: placebo; B: ACEIs; C: ARBs; D: CCBs.

Publication bias was verified by using comparison‐adjusted funnel plot. The symmetrical funnel plots showed no obvious publication biases were detected in Figure 9.

### 3.4 Withdrawal events related to cough between ACEIs and placebo

Ten studies reported participant discontinuation due to ACEI induced cough. Majority of participants who required discontinuation were from perindopril, ramipril, and enalapril groups. No severe outcome from cough was reported.

## 4 DISCUSSION

ACEIs are the cornerstone treatment of hypertension, heart failure, myocardial infarction, and cerebrovascular disease.^150^ This is the first meta‐analysis and network meta‐analysis of ACEI induced cough compared to placebo, ARB, and CCB. A common reported side effect of ACEI is cough, and it does not appear to be dose dependent.^151^ Ramipril is one of the most prescribed ACEIs and it ranked the second highest in causing cough among 11 ACEIs in this study. There is no significant difference of cough risk between each ACEI. Ramipril has six times higher risk of cough compared with placebo (95% CI: 2.61‐12.88) and 3.2 times (95% CI: 2.9‐3.53) with ARB. A large study, ONTARGET^152^ with over 25,000 participants, showed that the ramipril group resulted in higher treatment discontinuation due to cough when compared to telmisartan, an ARB (4.2% vs. 1.1%, *p* < .001). Our meta‐analysis results on ACEI versus ARB are similar to the ONTARGET study.

While captopril ranked the second least risk of causing cough compared to placebo (RR 3.11, 95% CI: 0.10–95.88), it is not statistically significant, and the analysis consisted of only one study on captopril with a sample size of 55. As a result, the confidence interval was extremely wide. Captopril was the first ACEI approved for use in 1980. Unlike most ACEIs captopril is one of the few ACEIs that is not a prodrug^153^ and it is well absorbed with a very short half‐life which requires administration three times a day.^154^ Due to its quick onset of action, it causes postural hypotension,^155^ captopril had been replaced by the newer ACEIs with longer half‐life, which requires once to twice a day administration.^155^, ^156^ Other commonly prescribed ACEIs such as perindopril and enalapril have 3.18 times, and 2.9 times risk of developing cough respectively, compared to placebo with statistical significance, but they are very similar to other ACEIs. Benazepril ranked top five in causing cough, however, it is not statistically significant compared with placebo or other ACEIs. The combined cough events caused by the ACEIs group ranked the highest when compared with other five groups. ACEIs performed 2.24 times versus placebo, 3.2 times versus ARBs and 6.5 times versus CCBs respectively. These risks are very similar to the risks in the individual meta‐analyses. This confirmed that the network meta‐analysis resulted in a good consistency and the network meta‐analysis was conducted satisfactorily.

The cough induced by ACEIs usually occurs within the first month of the first dose administration.^151^, ^157^ The symptoms resolve spontaneously after discontinuation of the ACEI within one to four weeks.^7^ ACEI induced cough occurs more frequently in females and nonsmokers.^9^, ^157^, ^158^, ^159^ Recent studies showed that individuals with polymorphisms in gene coding the bradykinin receptors, ACE (insertion/deletion), and aminopeptidase P which is responsible for the degradation of bradykinin are more susceptible to ACEI induced cough.^160^, ^161^, ^162^, ^163^

Our network meta‐analysis included commonly used ACEIs and offered valuable evidence that the risk of developing a cough is very similar in all ACEI as a class of agents. In addition, the results suggest that if a patient develops a dry cough from ACEIs, the best alternative is to switch to an ARB or CCB based on the patient's comorbidity. Ramipril is the last choice for the patients who are at risk of developing dry cough such as evidence of the gene mutation or living in poor air quality environment. In patients with risk of cough and the use of an ACEI is absolutely necessary, enalapril would be an option.

### 4.1 LIMITATION

In this study, we were unable to analyze dose related cough due to numerous different dosages used in the RCTs. Many studies had a small sample size, resulting the wide confidence interval which is important to judge the significant difference.

## 5 CONCLUSIONS

All ACEI has the similar risk of developing a cough. ACEI should be avoided in patients who have risk of developing cough, and an ARB or CCB is an alternative based on the patient's comorbidity.

## AUTHOR CONTRIBUTIONS

Yiyun Hu and Hoan Linh Banh conceived and conceptualized the research idea. Janice Y. Kung conducted comprehensive searches. Yiyun Hu and Hoan Linh Banh reviewed the search, performed the screening and full text assessment. Shuang Liu resolved any conflicts. Yiyun Hu and Hoan Linh Banh completed the quality assessment and data extraction. Ling Liang performed the data analyses, LL and Hoan Linh Banh interpreted the results. Ling Liang and Hoan Linh Banh contributed to the draft manuscript. All authors contributed to the final draft of the manuscript.

## CONFLICTS OF INTEREST STATEMENT

The authors declare no conflicts of interest.

## PATIENT CONSENT

This network meta‐analysis did not require patient recruitment. It does not require patient consent.

## PERMISSION TO REPRODUCE MATERIAL FROM OTHER SOURCES

All figures and tables are original and were created by the authors.

## CLINICAL TRIAL REGISTRATION

Not applicable.

## Data Availability

Authors have no data availability to share.

## SEARCH STRATEGIES.

**Table:**

Database		Search Strategy
**MEDLINE Ovid MEDLINE(R) ALL** 1946 to March 18, 2022	1.	exp Angiotensin‐Converting Enzyme Inhibitors/
	2.	(ACE inhibitor* or angiotensin converting enzyme inhibitor*).mp.
	3.	angiotensin converting enzyme antagonist*.mp.
	4.	angiotensin converting enzyme blocker*.mp.
	5.	dipeptidyl carboxypeptidase inhibitor*.mp.
	6.	benazepril*.mp.
	7.	Captopril/ or captopril.mp.
	8.	cilazapril*.mp. or exp CILAZAPRIL/
	9.	exp Enalapril/ or enalapril*.mp.
	10.	enalaprilat.mp. or exp ENALAPRILAT/
	11.	fosinopril*.mp. or exp FOSINOPRIL/
	12.	imidapril*.mp.
	13.	exp LISINOPRIL/ or lisinopril.mp.
	14.	moexipril*.mp.
	15.	perindopril*.mp. or exp PERINDOPRIL/
	16.	quinapril*.mp.
	17.	Ramipril/ or ramipril*.mp.
	18.	saralasin.mp. or exp SARALASIN/
	19.	Teprotide.mp. or exp TEPROTIDE/
	20.	trandolapril*.mp.
	21.	(alacepril or altiopril or ancovenin or ceranapril or ceronapril or deacetylalacepril or delapril or epicaptopril or fasidotril* or foroxymithine or gemopatrilat or idrapril or indolapril or libenzapril or moveltipril or omapatrilat or pentopril* or pivopril or rentiapril or s nitrosocaptopril or spirapril* or temocapril* or utibapril* or zabicipril* or zofenopril*).mp.
	22.	or/1‐21
	23.	Cough/
	24.	cough*.mp.
	25.	exp Bronchial Spasm/ or (bronchospasm* or bronchial spasm*).mp.
	26.	23 or 24 or 25
	27.	randomized controlled trial.pt.
	28.	clinical trial.pt.
	29.	randomi?ed.ti,ab.
	30.	placebo.ti,ab.
	31.	dt.fs.
	32.	randomly.ti,ab.
	33.	trial.ti,ab.
	34.	groups.ti,ab.
	35.	or/27‐34
	36.	animals/
	37.	humans/
	38.	36 not^36^ ^and^ ^37^
	39.	35 not 38
	40.	22 and 26 and 39
	41.	limit 40 to english language
**Embase Ovid Embase** 1974 to 2022 March 16	1.	exp dipeptidyl carboxypeptidase inhibitor/
	2.	(ACE inhibitor* or angiotensin converting enzyme inhibitor*).mp.
	3.	angiotensin converting enzyme antagonist*.mp.
	4.	angiotensin converting enzyme blocker*.mp.
	5.	dipeptidyl carboxypeptidase inhibitor*.mp.
	6.	benazepril*.mp.
	7.	captopril.mp.
	8.	cilazapril*.mp.
	9.	enalapril*.mp.
	10.	enalaprilat.mp.
	11.	fosinopril*.mp.
	12.	imidapril*.mp.
	13.	lisinopril.mp.
	14.	moexipril*.mp.
	15.	perindopril*.mp.
	16.	quinapril*.mp.
	17.	ramipril*.mp.
	18.	saralasin.mp. or exp saralasin/
	19.	Teprotide.mp.
	20.	trandolapril*.mp.
	21.	(alacepril or altiopril or ancovenin or ceranapril or ceronapril or deacetylalacepril or delapril or epicaptopril or fasidotril* or foroxymithine or gemopatrilat or idrapril or indolapril or libenzapril or moveltipril or omapatrilat or pentopril* or pivopril or rentiapril or s nitrosocaptopril or spirapril* or temocapril* or utibapril* or zabicipril* or zofenopril*).mp.
	22.	or/1‐21
	23.	exp coughing/
	24.	cough*.mp.
	25.	exp bronchospasm/ or (bronchospasm* or bronchial spasm*).mp.
	26.	23 or 24 or 25
	27.	Randomized controlled trial/ or Controlled clinical study/ or randomization/ or intermethod comparison/ or double blind procedure/ or human experiment/
	28.	(random$ or placebo or (open adj label) or ((double or single or doubly or singly) adj (blind or blinded or blindly)) or parallel group$1 or crossover or cross over or ((assign$ or match or matched or allocation) adj5 (alternate or group$1 or intervention$1 or patient$1 or subject$1 or participant$1)) or assigned or allocated or (controlled adj7 (study or design or trial)) or volunteer or volunteers).ti,ab.
	29.	(compare or compared or comparison or trial).ti.
	30.	((evaluated or evaluate or evaluating or assessed or assess) and (compare or compared or comparing or comparison)).ab.
	31.	or/27‐30
	32.	(random$ adj sampl$ adj7 (cross section$ or questionnaire$1 or survey$ or database$1)).ti,ab. not (comparative study/ or controlled study/ or randomi?ed controlled.ti,ab. or randomly assigned.ti,ab.)
	33.	Cross‐sectional study/ not (randomized controlled trial/ or controlled clinical study/ or controlled study/ or randomi?ed controlled.ti,ab. or control group$1.ti,ab.)
	34.	(((case adj control$) and random$) not randomi?ed controlled).ti,ab.
	35.	(Systematic review not (trial or study)).ti.
	36.	(nonrandom$ not random$).ti,ab.
	37.	Random field$.ti,ab.
	38.	(random cluster adj3 sampl$).ti,ab.
	39.	(review.ab. and review.pt.) not trial.ti.
	40.	“we searched”.ab. and (review.ti. or review.pt.)
	41.	update review.ab.
	42.	(databases adj4 searched).ab.
	43.	(rat or rats or mouse or mice or swine or porcine or murine or sheep or lambs or pigs or piglets or rabbit or rabbits or cat or cats or dog or dogs or cattle or bovine or monkey or monkeys or trout or marmoset$1).ti. and animal experiment/
	44.	Animal experiment/ not (human experiment/ or human/)
	45.	or/32‐44
	46.	31 not 45
	47.	22 and 26 and 46
	48.	limit 47 to english language
**CINAHL**	S1	(MH “Angiotensin‐Converting Enzyme Inhibitors+”)
	S2	“ACE inhibitor*” or “angiotensin converting enzyme inhibitor*”
	S3	“angiotensin converting enzyme antagonist*”
	S4	“angiotensin converting enzyme blocker*”
	S5	“dipeptidyl carboxypeptidase inhibitor*”
	S6	“benazepril*”
	S7	(MH “Captopril+”) or “captopril”
	S8	“cilazapril*”
	S9	(MH “Enalapril+”) or “enalapril*”
	S10	(MH “Enalaprilat”) or enalaprilat
	S11	(MH “Fosinopril”) or “fosinopril*”
	S12	“imidapril*”
	S13	(MH “Lisinopril+”) or “lisinopril”
	S14	“moexipril*”
	S15	(MH “Perindopril”) or “perindopril*”
	S16	“quinapril*”
	S17	“ramipril*”
	S18	“saralasin”
	S19	“Teprotide”
	S20	(MH “Trandolapril+”)
	S21	alacepril or altiopril or ancovenin or ceranapril or ceronapril or deacetylalacepril or delapril or epicaptopril or fasidotril* or foroxymithine or gemopatrilat or idrapril or indolapril or libenzapril or moveltipril or omapatrilat or pentopril* or pivopril or rentiapril or “s nitrosocaptopril” or spirapril* or temocapril* or utibapril* or zabicipril* or zofenopril*
	S22	S1 OR S2 OR S3 OR S4 OR S5 OR S6 OR S7 OR S8 OR S9 OR S10 OR S11 OR S12 OR S13 OR S14 OR S15 OR S16 OR S17 OR S18 OR S19 OR S20 OR S21
	S23	(MH “Cough”)
	S24	cough*
	S25	(MH “Bronchial Spasm”)
	S26	bronchospasm* or “bronchial spasm*”
	S27	S23 OR S24 OR S25 OR S26
	S28	(MH (randomized controlled trials OR double‐blind studies OR single‐blind studies OR random assignment OR pretest‐posttest design OR cluster sample) OR TI (randomised OR randomized) OR AB random* OR TI trial OR ((MH (sample size) AND AB (assigned OR allocated OR control))) OR MH (placebos OR crossover design OR comparative studies) OR AB ((control W5 group) OR (cluster W3 RCT) OR PT (randomized controlled trial))) NOT ((MH animals+ OR MH (animal studies) OR TI (animal model*)) NOT MH (human))
	S29	S22 AND S27 AND S28
	S30	S22 AND S27 AND S28 (Limiters: English Language)
**Cochrane**	#1	[mh “Angiotensin‐Converting Enzyme Inhibitors”]
**Library**	#2	ACE inhibitor* or angiotensin converting enzyme inhibitor*
(Wiley)	#3	angiotensin converting enzyme antagonist*
Cochrane Reviews, Trials	#4	angiotensin converting enzyme blocker*
	#5	dipeptidyl carboxypeptidase inhibitor*
	#6	benazepril*
	#7	[mh ^Captopril] or captopril
	#8	[mh CILAZAPRIL] or cilazapril*
	#9	[mh Enalapril] or enalapril*
	#10	[mh ENALAPRILAT] or enalaprilat
	#11	[mh FOSINOPRIL] or fosinopril*
	#12	imidapril*
	#13	[mh LISINOPRIL] or lisinopril
	#14	moexipril*
	#15	[mh PERINDOPRIL] or perindopril*
	#16	quinapril*
	#17	[mh ^Ramipril] or ramipril*
	#18	[mh SARALASIN] or saralasin
	#19	[mh TEPROTIDE] or Teprotide
	#20	trandolapril*
	#21	alacepril or altiopril or ancovenin or ceranapril or ceronapril or deacetylalacepril or delapril or epicaptopril or fasidotril* or foroxymithine or gemopatrilat or idrapril or indolapril or libenzapril or moveltipril or omapatrilat or pentopril* or pivopril or rentiapril or s nitrosocaptopril or spirapril* or temocapril* or utibapril* or zabicipril* or zofenopril*
	#22	{OR #1‐#21}
	#23	[mh ^Cough]
	#24	cough*
	#25	[mh “Bronchial Spasm”] or bronchospasm* or bronchial NEXT spasm*
	#26	{OR #23‐#25}
	#27	#22 AND #26
**Scopus**		TITLE‐ABS‐KEY ({ACE inhibitor*} OR {angiotensin converting enzyme inhibitor*} OR {angiotensin converting enzyme antagonist*} OR {angiotensin converting enzyme blocker*} OR {dipeptidyl carboxypeptidase inhibitor*} OR benazepril* OR captopril OR cilazapril* OR enalapril* OR enalaprilat OR fosinopril* OR imidapril* OR lisinopril OR moexipril* OR perindopril* OR quinapril* OR ramipril* OR saralasin OR teprotide OR trandolapril* OR alacepril OR altiopril OR ancovenin OR ceranapril OR ceronapril OR deacetylalacepril OR delapril OR epicaptopril OR fasidotril* OR foroxymithine OR gemopatrilat OR idrapril OR indolapril OR libenzapril OR moveltipril OR omapatrilat OR pentopril* OR pivopril OR rentiapril OR {s nitrosocaptopril} OR spirapril* OR temocapril* OR utibapril* OR zabicipril* OR zofenopril*) AND TITLE‐ABS‐KEY (cough* OR bronchospasm* OR {bronchial spasm*}) AND TITLE‐ABS‐KEY ({Clinical‐trial} OR {controlled‐trial} OR randomi* OR randomly OR (random W/4 (allocat* OR distribut* OR assign*)) OR {placebo} OR {trial} OR {groups} OR {subgroups}) OR TITLE (rct) AND (LIMIT‐TO (LANGUAGE, “English”)) AND (EXCLUDE (DOCTYPE, “no”) OR EXCLUDE (DOCTYPE, “sh”) OR EXCLUDE (DOCTYPE, “le”) OR EXCLUDE (DOCTYPE, “ed”) OR EXCLUDE (DOCTYPE, “ch”))
		Excluded Document Type: Note, Short survey, Letter, Editorial, Book Chapter
**Google Scholar**		(ACE inhibitors OR “angiotensin converting enzyme”) AND cough
